# Determination of Relative Density and Degree of Saturation in Mineral Soils Based on In Situ Tests

**DOI:** 10.3390/ma14226963

**Published:** 2021-11-17

**Authors:** Simon Rabarijoely, Mariusz Lech, Marek Bajda

**Affiliations:** Department of Geotechnics, Institute of Civil Engineering, Warsaw University of Life Sciences, Nowoursynowska 159 St., 02-776 Warsaw, Poland; mariusz_lech@sggw.edu.pl (M.L.); marek_bajda@sggw.edu.pl (M.B.)

**Keywords:** geotechnical engineering, new nomogram chart

## Abstract

Based on the results of dynamic probing (DP), time-domain reflectometry (TDR/MUX/MPTS), resistivity cone penetration tests (RCPT), Marchetti dilatometer tests (DMT), and seismic dilatometer tests (SDMT), it is possible to develop a relationship to calculate the relative density (*D_r_*) and degree of saturation (*S_r_*) of selected sandy soils. Compiled databases from documented research points for selected sandy soils were used to construct and develop direct correlations between the measured pressures *p*_0_ and *p*_1_ from DMT and shear wave velocity (*V_s_*) from SDMT, along with pore water pressures (*u*_0_) and atmospheric pressure (Pa). The results allowed us to make a preliminary prediction when evaluating the parameters. Further, they allowed limiting the use of an additional device, especially in the case of multilayer heavy preconsolidated subsoils. Moreover, soil physical and mechanical characteristics (temperature, humidity, pressure, swelling, salinity) measured from TDR/MUX/MPTS (laboratory/field-operated meter for simultaneous measurements of soil moisture, matric potential, temperature, and salinity—bulk electrical conductivity) were assessed. The main achievement of this paper is the original proposal of using a new nomogram chart to determine the relative density and degree of saturation based on DMT and SDMT tests.

## 1. Introduction

In accordance with the applicable construction law (Eurocode 7), each project applying for a building permit should include, depending on the needs, the results of geological and engineering research. This documentation consists of the developed results of field and laboratory tests. The advantage of field tests is the fact that they take place in the natural environment, which is often difficult to recreate in laboratory conditions. Probing is an example of such research work carried out to determine the ground condition. Among numerous available methods, dynamic probing tests (DP), cone penetration tests (CPT), and dilatometer Marchetti tests (DMT) are commonly applied in leading research centers. In order to reduce the necessity to use various types of equipment, methods of field research are being sought for to enable the interpretation of the obtained results in a wide range [1,2,3,4,5]. One of the field tests that meets this requirement is the Marchetti dilatometer test [6], whose use in the world is significantly increasing.

**Figure 1:**
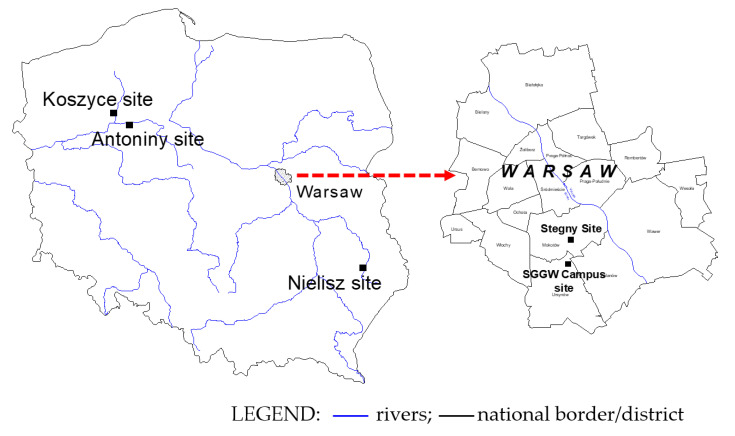
Location of the test sites in Poland.

The greatest advantage of dilatometer testing is its quick and relatively simple measurement, on the basis of which it is possible to determine the soil parameters. Interpretation of geotechnical parameters is based on the use of empirical relationships related to pressure values measured directly in the field. This article is based on this type of in situ research. Based on the results of DP, DMT, SDMT, and RCPT tests carried out in the Antoniny, Koszyce, and Nielisz sites with organic subsoils under embankment, and the Stegny and WULS-SGGW campus sites with a sandy soil layer (Figure 1), new relationships were determined to assess the parameters describing the state of selected mineral soils. Since cohesive and organic soils were not taken into account to develop the formulas for determining the parameters of *D_r_* and *S_r_*, these parameters were not included in their physical properties in this article.

This paper presents the test results of mineral and organic subsoils obtained from the following sites (WGS-84): Antoniny (WGS-84: 53.06742, 17.07214), Koszyce (WGS-84: 53.17045, 16.74924), Nielisz (WGS-84: 50.80396, 23.03075), Stegny (WGS-84: 52.18215, 21.04864), and the WULS-SGGW campus (WGS-84: 52.16245, 21.03838).

The Antoniny test embankment was designed and tested in the frame of cooperation between the Department of Geotechnical Engineering SGGW and the Swedish Geotechnical Institute (SGI). The physical properties of the soils at the Koszyce, Nielisz, Stegny, and WULS-SGGW campus test sites were determined as part of a research program conducted at these sites in previous years. 

The Antoniny embankment and Koszyce test dam are located in the Ruda river valley. A layer of soft organic soils was discovered in the sublayer of both objects. The organic soils are Quaternary deposits of an oxbow lake. The thickness of organic soils in this region generally exceeds 10 m and locally even 20 m. Dense sand occurs under the organic soils. The Nielisz site is located in eastern Poland in the Wieprz river valley in Lublin Province. The layer of soft subsoil has a thickness of 3 to 5 m; the soils are slightly preconsolidated. Two layers of organic subsoil were distinguished at the Nielisz site. The Stegny and SGGW campus sites are located in the southern part of Warsaw, where a few sedimentation cycles, from sands to clays, were observed in vertical succession. The entire complex of Pliocene clays comprises clays, silty clays (60–70%), silts (10–25%), and sands (10–20%). 

The index properties of all investigated mineral and organic soils are presented in Table 1.

## 2. Literature Review

### 2.1. Methodology and Interpretation of Dilatometer Test Results

Over 46 years ago, Prof. Silvano Marchetti designed and built the first dilatometer at L’Aquila University in Italy; the design and principles of soil research were presented by him in 1975 during the American Society of Civil Engineers (ASCE) conference in Raleigh [7]. DMT tests consist of measuring the gas pressure acting on the diaphragm of a dilatometer blade at selected subsoil depths (Figure 1). In soil tests, two pressures are usually measured (A and B); they force the center of the membrane to move 0.05 mm to the ground (reading A) and deflect the center of the membrane towards the ground by approximately 1.05 mm (reading B). To extend the dilatometer testing, pressure measurements are sometimes taken as the membrane returns to ground contact (C reading). Readings A, B, and C are corrected for the inertia of the diaphragm and marked as *p*_0_, *p*_1_, and *p*_2_, respectively. Pressures *p*_0_ and *p*_1_ and the value of the vertical effective stress $\sigma_{v0}^{\prime }$ are used to determine the following dilatometer indexes: material index *I_D_*, horizontal stress index *K_D_*, and dilatometer modulus *E_D_* [8,9,10].

(1) $$p_{0}=1.05\left(A-Z_{M}+\Delta A\right)-0.05\left(B-Z_{M}-\Delta B\right),\;\left(\mathrm{MPa}\right),$$

- The 1.10 mm corrected pressure reading in DMT *p*_1_:

(2) $$p_{1}=B-Z_{M}-\Delta B,\;\left(\mathrm{MPa}\right),$$

- Corrected third reading in DMT *p*_2_:

(3) $$p_{2}=C-Z_{M}-\Delta A\;\left(\mathrm{MPa}\right),$$

- Material index *I_D_*:

(4) $$I_{D}\left(-\right)=f\left(A,\;B,\;u_{0}\right)=\frac{P_{1}-P_{0}}{P_{0}-u_{0}},$$

- Horizontal stress index *K_D_*:

(5) $$K_{D}\left(-\right)=f\left(A,\;u_{0},\sigma_{v0}^{\prime },B\right)=\frac{p_{0}-u_{0}}{\sigma_{v0}^{\prime }},$$

- Dilatometer modulus *E_D_*:

(6) $$E_{D}\left(\mathrm{MPa}\right)=f\left(A,\;B\right)=34.7\cdot \left(P_{1}-P_{0}\right),$$

- Pore pressure index *U_D_*:

(7) $$U_{D}\left(-\right)=f\left(A,\;C,\;u_{0},B\right)=\frac{P_{2}-u_{0}}{P_{0}-u_{0}},$$

where *p*_0_—pressure reading *A* corrected for *Z_m_* and Δ*A* membrane stiffness at 0.05 mm expansion, and 0.05 mm expansion itself, to estimate the total soil stress acting normal to the membrane immediately before its expansion into the soil (0.00 mm expansion);

*p*_1_—pressure reading *B* corrected for *Z_m_* and Δ*B* membrane stiffness at 1.10 mm expansion to give the total soil stress acting normal to the membrane at 1.10 mm membrane expansion;

*p*_2_—pressure reading *C* corrected for *Z_m_* and Δ*A* membrane stiffness at 0.05 mm expansion and used to estimate pore water pressure;

$\sigma_{v0}^{\prime }$—pre-insertion in situ overburden stress;

*u*_0_—pore water pressure acting in the center of the membrane before insertion of the DMT blade (often assumed as hydrostatic below the groundwater table);

*Z_m_*—gage pressure deviation from zero when vented to atmospheric pressure (offset used to correct pressure readings to the true gage pressure).

### 2.2. Existing In Situ Methods for Determining Relative Density and Degree of Saturation in Non-Cohesive Soils

By definition, relative density is a parameter that characterizes non-cohesive soils. It is the ratio of soil compaction in the natural state to the highest possible compaction of a specific soil. There are two types of dependence for determining relative density (*D_r_*) on the basis of DMT. The first is the relationship presented by Reyna and Chameau (1991) [11], and Mayne (2001) [12], and it depends on the horizontal pressure index (*K_D_*) from DMT tests. The second relationship was described by Marchetti (1992) [13]; it is a function of the dilatometer blade resistance (*q_D_*) and effective vertical stress ($\sigma_{v0}^{\prime }$). In the literature, there are many formulas to determine relative density from in situ studies [11,12,13]. These dependencies are presented below:

(8) $$D_{r}=-1.082+0.204\cdot {\left(\frac{q_{D}}{\sigma_{v0}^{\prime }}\right)}^{0.4},$$

where *D_r_* is relative density (as decimal); $\sigma_{v0}^{\prime }$ is effective geostatic stress (kPa); and *q_D_* is wedge resistance.

(9) $$D_{r}={\left[\frac{1}{40\cdot \left(K_{D}-1\right)}+\frac{1}{120}\right]}^{-1.0},$$

For normally consolidated (NC) uncemented sands, the recommended equation for the relative density (*D_r_*) of non-cohesive soils is shown in Formula (8) [11], where parameter *D_r_* is related to *K_D_* from DMT research. This correlation is influenced by the additional *K_D_*−*D_r_* data points (also in Formula (9)) obtained by Tanaka and Tanaka (1998) [14] from the Ohgishima and Kemigawa sites, where parameter *D_r_* was established on the basis of high-quality samples collected using the freezing method. In fractured sands in the range of preconsolidated stress (OC) (Formula (10)), parameter *D_r_* will be overestimated because part of the *K_D_* value is due to the influence of preconsolidation and cementation. At present, it is difficult to clearly assess the value of parameter *D_r_*.

(10) $$D_{r}=100\cdot {\left(\frac{K_{D}-1}{7}\right)}^{0.5},$$

Formulas (8)–(10) do not sufficiently describe a satisfying (small cyclic shear stress factor) state of non-cohesive soils in a wide range of interpretations of the saturation state with the shear wave velocity propagation for a given soil medium, and their influence on the compressibility of the air–water mixture filling the pores and on the compressibility of the soil skeleton. Therefore, later in this article, a decision is made to develop a new relationship for determining the degree of relative density of non-cohesive soils based on vs. obtained from SDMT tests.

## 3. Materials and Methods

### 3.1. Material

**Figure 2:**
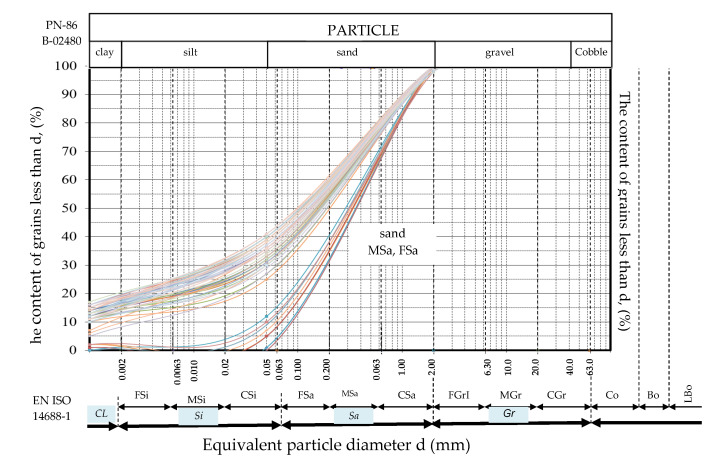
Grain size distribution curve obtained from laboratory tests for the sandy soils from the described sites.

**Figure 3:**
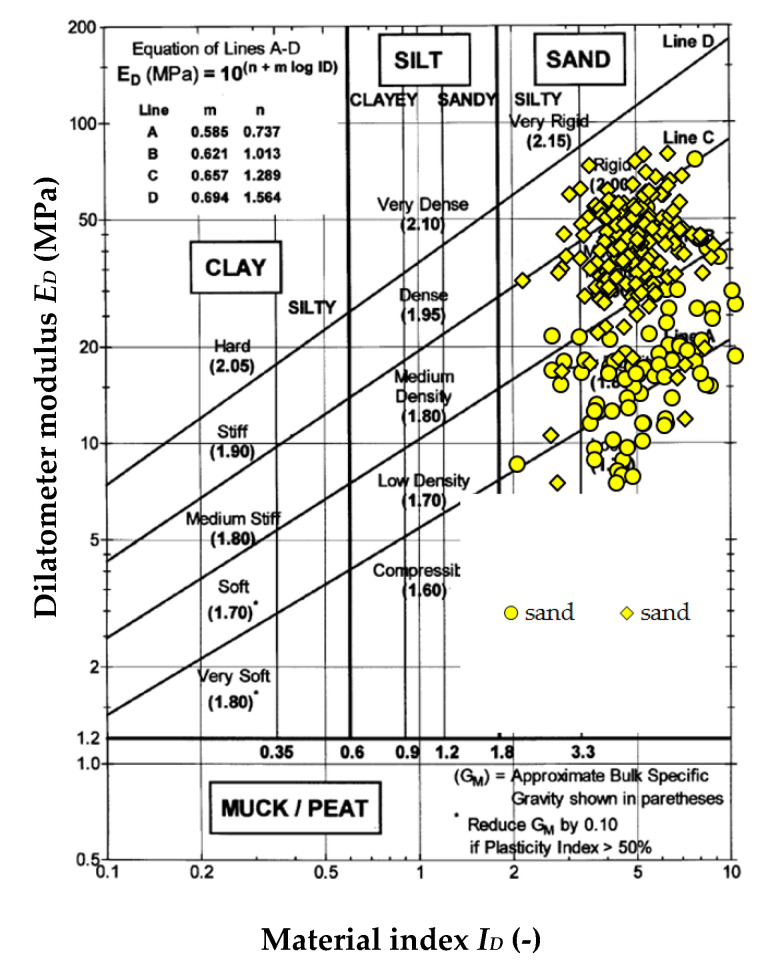
Chart for estimating unit weight (normalized to γ_w_) of sands in the Antoniny, Koszyce, Nielisz, Stegny, and WULS-SGGW campus test sites [19].

This paper contains the test results of sands located in the subsoil and in embankments on the test sites that were presented in Section 1, where a laboratory and field testing program was carried out under and outside of the main dam embankment [15,16,17,18]. This research was carried out according to the Casagrande method modified by Prószyński. Soil grains in the range from 0.001 to 0.1 mm were collected using the areometric method, and the remaining fractions in the range above 0.1 mm were collected by the sieve method. The results of fraction testing from 0.001 to 0.1 mm were read together with smaller percent passing, and above 0.1 mm with larger percent retaining. After dividing the range of a particular fraction into clay, silt, sand, gravel, and cobble (the sum of these fractions amounts to 100%), the soil type was determined according to PN-86 B-02480. For the embankment layer at the Antoniny site, the clay fraction content was 0%, the clay fraction ranged from 2 to 3%, the sand fraction ranged from 88 to 91%, and the gravel fraction was about 7%. At the Koszyce site, the clay fraction content was 0%, the clay fraction ranged from 2 to 3%, the sand fraction ranged from 89 to 92%, and the gravel fraction was about 5%. At the Nielisz site, the clay fraction content was 0%, the clay fraction ranged from 3 to 5%, the sand fraction ranged from 87 to 92%, and the gravel fraction was about 2%. At the SGGW campus site, the clay fraction content was 1%, the clay fraction was 4%, the sand fraction ranged from 88 to 95%, and the gravel fraction was 0%. At the Stegny site, the clay fraction content was 0%, the clay fraction was 3%, the sand fraction was 92%, and the gravel fraction was about 5%. The grain size distribution curve obtained from laboratory tests for mineral soils from the described sites is presented in Figure 2. The index properties of mineral soils in the Antoniny, Koszyce, Nielisz, Stegny, and WULS-SGGW campus test sites are presented in Table 2. Figure 3 presents the diagram chart proposed by Marchetti and Crapps for the analyzed sites [19].

### 3.2. Laboratory Test with TDR/MUX/mpts Meter

**Figure 4:**
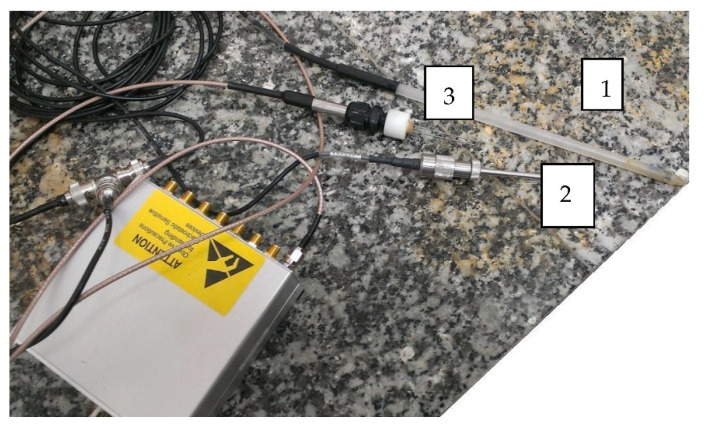
TDR/MUX/mpts meter with the following sensors: 1. temperature, 2. suction pressure, and 3. moisture and salinity (Laboratory of the Department of Geotechnics, WULS-SGGW).

The laboratory TDR/MUX/mpts meter [20] was adapted to measure the following parameters: volumetric moisture expressed as percentage value using the LP/MS probe; soil salinity with the LP/MS probe, expressed in S/m; parent pressure inside the soil sample with the LP/p probe, expressed in mbar; and temperature inside the soil sample with the LP/t probe, expressed in °C. All tests were performed in the Laboratory of the Department of Geotechnics of the WULS–SGGW (Figure 4).

Each of the sensors was placed in a previously prepared soil sample. The tests were carried out on 2 soil samples: silty sand and silty clay. Before testing, the soil samples were compacted and placed in a steel cylinder with holes for sensor placement. Then, the Lp probe was prepared, which required specialized calibration. Preparation of the meter included the following steps: 1. Inserting the LP/t sensor (temperature sensor) into distilled water, while the LP/p sensor (pressure sensor) should be deaerated and put into a vessel with distilled water. 2. Turning on the readout logging program and defining the names of the saved files. In the program start window, the appropriate channel in which the program will work should be selected. 3. Calibrating the sensors, inserting the moisture probe into the calibrator, and then into distilled water, so that the water reaches the sensor cap, waiting for 2 min, and then following the instructions on the computer monitor. 4. Starting the readings. The probes should be placed in the ground in order not to damage the ground structure.

**Figure 5:**
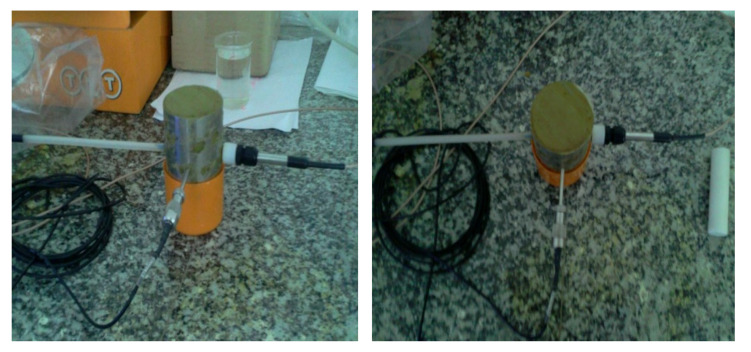
A sample of silty sand with inserted sensors (Laboratory of the Department of Geotechnics, WULS–SGGW).

The first to be tested was a silty sand sample, which was pressed into a steel cylinder that was previously weighed on a laboratory scale. Then, the sensors of the LP probe were pressed, and everything was placed on the scale. The following results were obtained: cylinder weight = 344.50 g; weight of cylinder + silty sand = 801.40 g; total weight = 858.85 g; weight of analyzed silty sand = 456.90 g. The prepared soil sample with the inserted sensors is shown in Figure 5.

**Figure 6:**
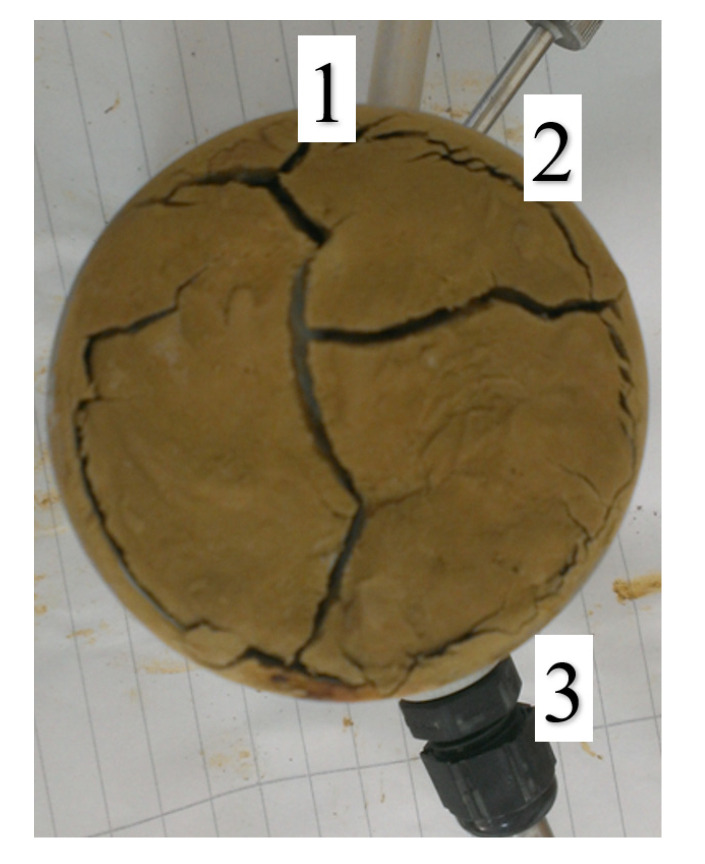
Soil sample with cracks and probe sensors: 1. temperature, 2. suction pressure, and 3. moisture and salinity (Laboratory of the Department of Geotechnics, WULS–SGGW).

The volume of the test sample was also determined (237.6 cm^3^), and the sample humidity was calculated. The reading was set to 60 s. After 20 days of the analysis, the “drying” of the sample was completed, and the sample was placed on the laboratory scale: weight of total sample after “drying” = 771.85 g; weight of silty sand after drying = 369.90 g. After weighing, 100 g of water was added to the rehydrate, and the sample returned to its original condition. After 8 days of contact of the sample with water, the sandy clay analysis was completed and the soil was reweighed: final weight of soil with sensors and cylinder = 819.30 g; weight of hydrated clay = 474.80 g; weight of the remaining water = 16.80 g. After drying the sample, cracks in the sample were noticed, indicating shrinkage of the tested soils. Soil shrinkage is shown in Figure 6.

Finally, the following dependencies were plotted: soil sample temperature vs. experiment duration; sample moisture vs. time; salinity vs. time; soil sample swelling due to humidity. After the test, the test soil was removed, the probe tips and the metal cylinder were cleaned, and then a clay sample was placed in the cylinder. The clay tests were carried out in the same way as the silty sand tests.

### 3.3. Estimation Theory

**Figure 7:**
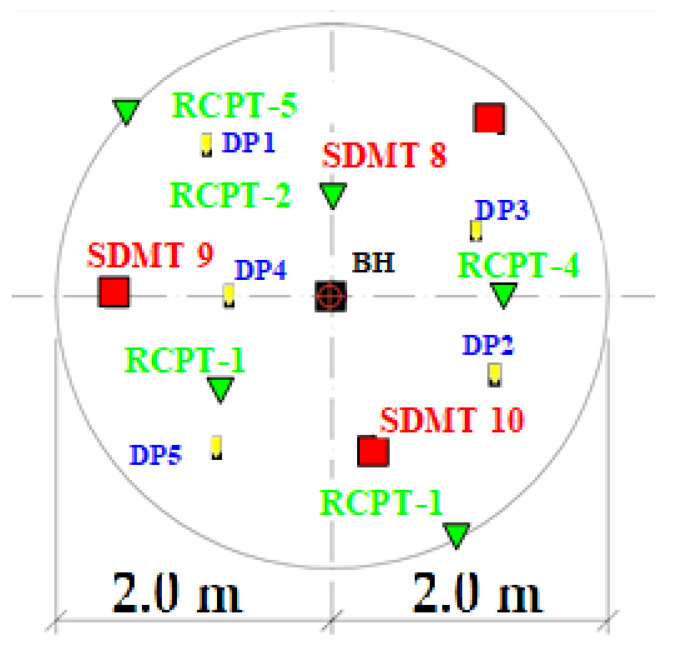
Location of DH, SDMT, and RCPT test point positions—the Stegny site.

For the determination of parameters *D_r_* and *S_r_* based on DMT tests, parameters *p*_0_, *p*_1_, *u*_0_, and $\sigma_{v0}^{\prime }$ were taken into account in the statistical analysis. These quantities can be treated as random variables. A total of 48 DMT tests from the sand layer were used, with a count of 10 to 35 measurements (depth profiles), with a total of 255 measurements. The distribution of the tested random variables was checked. For example, at the Stegny test site, a set of investigations was performed, which consisted of a borehole (BH), 10 DMT soundings (including 3 SDMT), 5 RCPT soundings, and 5 dynamic DP soundings. Surveys were located in the immediate vicinity of the borehole (within two meters): SDMT—3 units, RCPT—5 units, DP—5 units, and the results of these surveys were used to create a database (see attached Figure 7).

Measurements in the SDMT survey were performed every 20 cm, which gave a total of 17 measurements in the profile from 1 to 4.2 m. Each of these measurements was paired with a result from the DP dynamic tests or RCPT—this returned a total number of 3 (SDMT) × 17 × 5 (DP), i.e., 255, measurements.

The remaining 7 DMTs from the Stegny test site and studies from the Antoniny, Koszyce, Nielisz, and SGGW campus sites were used to verify the proposed relationships (*D_r_* and *S_r_*). In the case of most studies, i.e., random samples in a statistical sense, there was no reason to reject the hypothesis of normality (Shapiro–Wilk tests suitable for small samples were applied; they are available, e.g., in the Statgraphics statistical software package). No other distribution was found to which the tested quantities complied [21,22,23].

The purpose of determining new formulas for calculating relative density (*D_r_*) is to derive the dependencies closely related to the parameters of the measurements with the dynamic DP probe. Apart from the parameters of the DMT tests, the results of RCPT were also used. Several stages to determine the authors’ dependencies calculating the subsoil state differed for the given parameters. This is due to the fact that we had different data for specific sites. For determining the relative density (*D_r_*) on the basis of the dependence of DP and RCPT results with the transition to DMT, consisting of performing all the calculations carried out in this work, the solver module was used. It is an addition that extends the functionality of MS Office after it is imported. This function is most often used for linear programming including the modeling and optimization of any type of decision problem. This linear programming should be based on the creation of a reality model, in which the objective function is an important element, the value of which is subordinated to a specific profitability criterion (max/min). The solution of the function is assisted by the use of variable coefficients (for relative density determined in this paper, *α*_0_, *α*_1_, and *α*_2_ were assumed), due to which maximum or minimum values are achieved.

Observation of the obtained results shows that there is a strong relationship between parameter *D_r_* and dilatometer pressures, i.e., *p*_0_ and *p*_1_, and shear wave velocity, as well as *u*_0_ and $\sigma_{v0}^{\prime }$: *D_r_* = *f* (*p*_0_, *p*_1_, *u*_0_, $\sigma_{v0}^{\prime }$). For non-cohesive soils in the studied sites, a formula was proposed on their basis, and the lowest value of the mean square relative deviation was calculated for them. The formula that was used for the calculations is our original proposal. The summarized test results, i.e., *p*_0_ and *p*_1_ vs. values from DMT and *u*_0_ tests, allowed determining the formula for relative density (*D_r_*) in the following form:

(11) $$D_{r}=f\left(\alpha_{0},\;\alpha_{1},\;\alpha_{2},\;p_{0},\;p_{1},\;V_{s},\;u_{0},\;\sigma_{v0}^{\prime }\right),$$

(12) $$D_{r}=\alpha_{0}\cdot {\left(\frac{p_{1}-u_{0}}{\sigma_{v0}^{\prime }}\right)}^{\alpha_{1}}\cdot {\left(\frac{V_{s}}{100}\right)}^{\alpha_{2}},$$

where *α*_0_, *α*_1_, and *α*_2_ are the coefficients. The formula was created dependent on variables $p_{0},\;p_{1},\;u_{0},\;\sigma_{v0}^{\prime },$ which belong to variable cells in the solver function. An additional column was added, the formula of which determines the percentage deviation between the result of the calculated relative density (*D_r_*) for a given depth and the value of the result obtained from DP tests. The given depth is the corresponding level of the measuring point at which the readings from both SDMT (DMT) and DP are recorded. These are the readings every 20 cm. The purpose of the calculations was to obtain the lowest possible mean square relative deviation *MSRD*. The values for the statistical compilation were calculated from the following formulas:

Maximal relative deviation:

(13) $$MRD\underset{i=1,2,\ldots ,m}{\max}\left|\frac{y_{i}-\tilde{y_{i}}}{y_{i}}\right|\cdot 100\%,$$

Mean square relative deviation:

(14) $$MSRD=\left[\sqrt{\frac{1}{m}\sum_{i=1}^{m}{\left(\frac{y_{i}-\tilde{y_{i}}}{y_{i}}\right)}^{2}}\right]\cdot 100\%,$$

The function was programmed in a way that allowed achieving the lowest possible target cell value. The results of the calculations are presented in Section 4 below. For non-cohesive soils located in the study area, i.e., in the “Stegny” and WULS-SGGW sites, and the data obtained from the dilatometer test, a formula was proposed, and the relative density was calculated.

As for the second parameter, which is the degree of saturation (*S_r_*), the results of dilatometer tests (DMT) were also used to determine it. Following the observations, there was a strong relationship between parameter *S_r_* and pressures *p*_0_ and *p*_1_, and parameters *u*_0_ and $\sigma_{v0}^{\prime }$: $S_{r}=f\left(p_{0},p_{1},u_{0},\sigma_{v0}^{\prime }\right)$. For non-cohesive soils located in the studied sites, a formula was proposed on their basis, and the lowest value of the mean square relative deviation was calculated. The formula on the basis of which the calculations were performed is also an original proposal of our team and attains the following form:

(15) $$S_{r}=f\left(\beta_{0},\;\beta_{1},\;p_{0},\;p_{1},\;u_{0},\;\sigma_{v0}^{\prime }\right),$$

(16) $$S_{r}=\beta_{0}\cdot {\left[\frac{\left(P_{0}-u_{0}\right)\left(P_{1}-u_{0}\right)}{\sigma_{v0}^{\prime }}\right]}^{\beta_{1}},$$

## 4. Test Results

**Figure 8:**
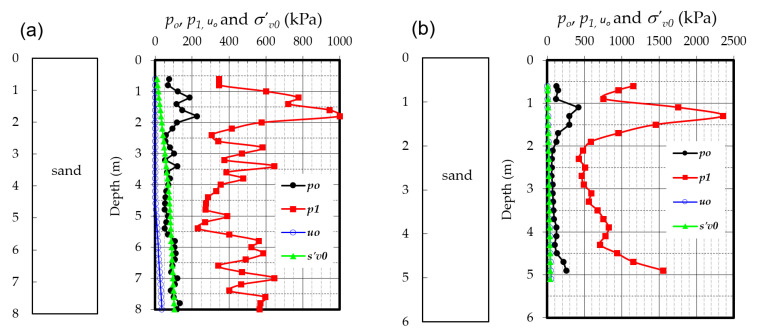

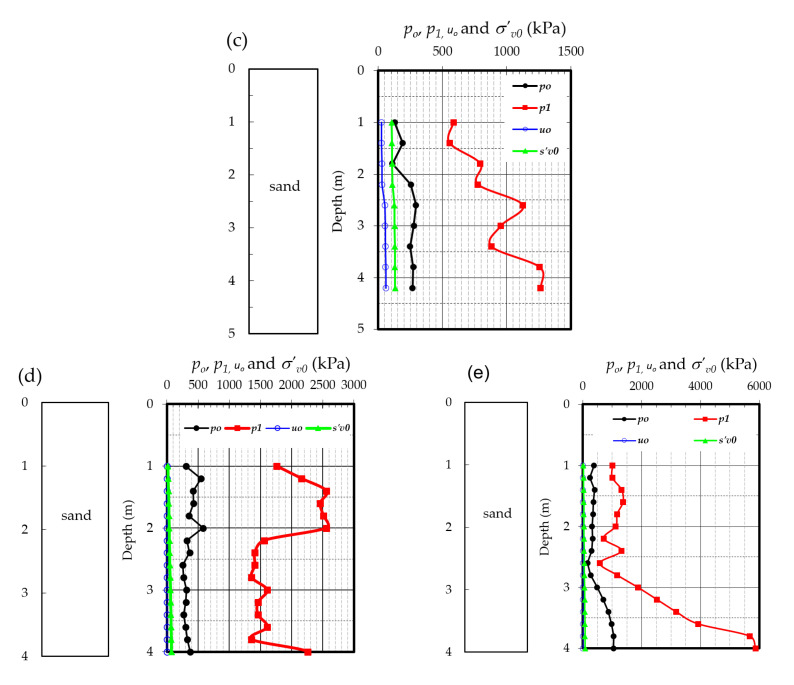
Profiles of *p*_0_, *p*_1_, and *p*_2_ from dilatometer (DMT) and hydrostatic pressure *u*_0_ tests for the: (**a**) Antoniny site; (**b**) Koszyce site; (**c**) Nielisz site; (**d**) Stegny site; and (**e**) WULS-SGGW campus site.

The DMT test results obtained for the Antoniny, Koszyce, Nielisz, Stegny, and WULS-SGGW campus sites are presented in Figure 8. They were taken into account in the construction of a new correlation for mineral and organic soils presented in the following chapter.

### 4.1. Estimation of Relative Density

**Figure 9:**
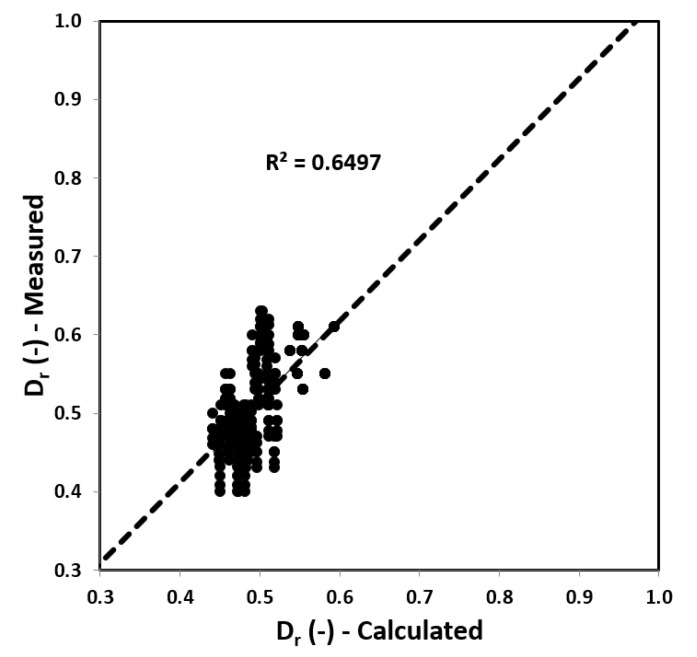
Summary of the values obtained from the DP tests and calculated using Formula (17) from the analyzed Stegny site.

Using the solver module, the values of the coefficients (*α*_0_, *α*_1,_ *α*_2_) were determined, where there is the lowest value of the mean square relative deviation for pressure, expressed in kPa. For the proposed formula for relative density (*D_r_*), the following statistical values were obtained (summarized in Figure 9 below). The obtained pattern is as follows:

(17) $$D_{r}=\alpha_{0}\cdot {\left(\frac{p_{1}-u_{0}}{\sigma_{v0}^{\prime }}\right)}^{\alpha_{1}}\cdot {\left(\frac{V_{s}}{100}\right)}^{\alpha_{2}},$$

where *α*_0_ = 0.35, *α*_1_ = 0.14, and *α*_2_ = 0.16.

**Figure 10:**
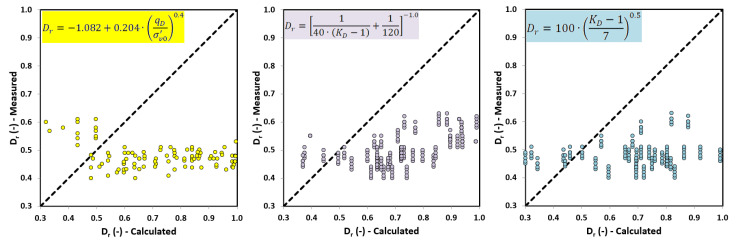
Values obtained from TDR tests and RCPT related to the values calculated from formulas (Equations (8)–(10)) based on DMT tests.

The values obtained from the DP test related to the values calculated based on Formulas (8)–(10) using *q_D_* and *K_D_* from DMT tests show a large range of the mean error results. The correlation between the values calculated (from Formulas (8)–(10)) and measured is presented in Figure 10.

From the chart, it can be seen that the values calculated using the formula correspond well with the values obtained from the DP tests. They behave predictably and fit into an overall trend line, while the DP test values have higher amplitudes. The relationship between the results obtained from DP tests and those determined on the basis of the Formula (17) is presented above.

Using the proposed dependencies to determine the relative density (*D_r_*) in the case of fine-grained soils, the compaction index *I_s_* can also be determined as follows using and modifying the formula developed by Pisarczyk (1975, 2015) [24,25,26] and the dependencies proposed herein:

(18) $$I_{S}=0.855+0.058\cdot \left[\;K_{D}^{*0.14}\cdot {\left(\frac{V_{s}}{100}\right)}^{0.16}\right],$$

where $K_{D}^{*}\left(-\right)=\frac{p_{1}-u_{0}}{\sigma_{v0}^{\prime }}$.

### 4.2. Estimation of the Degree of Saturation (S_r_)

**Figure 11:**
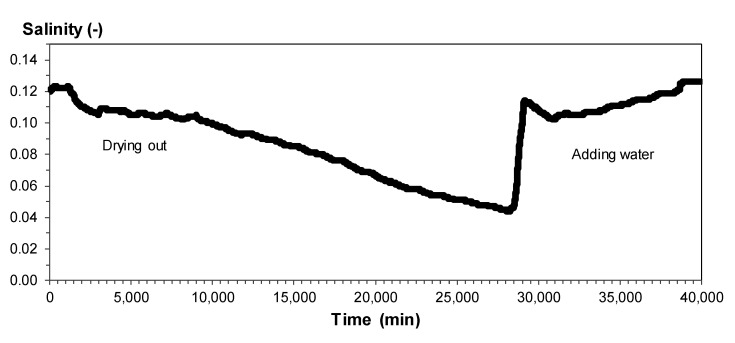
Time dependence of salinity for silty sand from depth level of 3.5 m.

**Figure 12:**
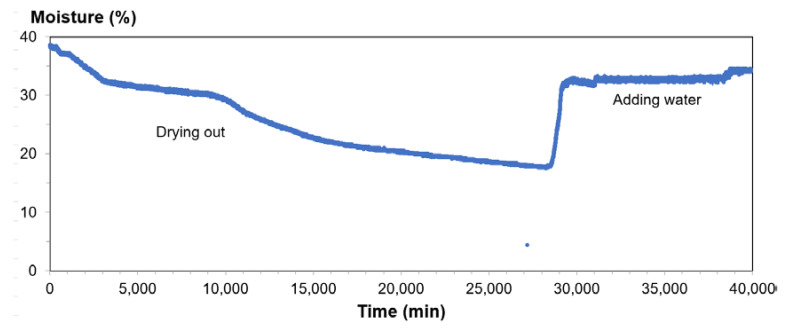
Time dependence of humidity for silty sand from depth level of 3.5 m.

**Figure 13:**
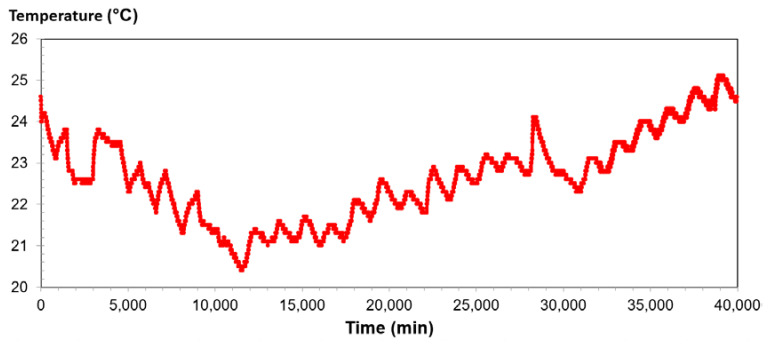
Relationship between temperature and time for silty sand from depth level of 3.5 m.

The results of the TDR/MUX/mpt tests and RCPT were used to determine parameter *S_r_*. The following graphs were obtained during the tests: temperature dependence on time; soil swelling dependence on time; time dependence of humidity; and dependence of soil salinity on time. Graphs for a silty sand sample are presented below (Figure 11, Figure 12 and Figure 13). In these figures, the following observations can be made:

- When examining the dependence of salinity on time, the amount of salinity decreases with soil drying but increases with the addition of water. The minimum salinity value is 0.044, while the maximum value is 0.126.
- During the analysis of the time dependence of humidity, the minimum value was obtained with complete drying of the soil; this value was 17.6%, while the highest was 38.2%.
- When analyzing the dependence of temperature on time, daily temperature fluctuations may be noticed; the lowest was 20.4 °C, while the highest was 25.1 °C.

**Figure 14:**
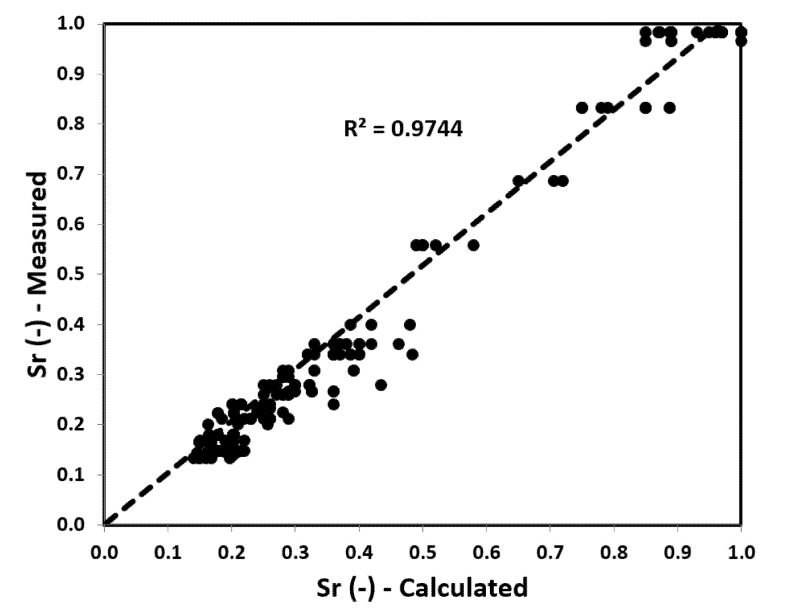
*S_r_* values obtained from TDR tests and RCPT related to the calculated *S_r_* values from the proposed formula based on DMT tests.

According to the diagram (Figure 14), the concordance between the parameter calculated on the basis of DMT and the parameter obtained from the TDR and RCPT results is high (*MSRD* = 12%, *MRD* = 22%). These values provide a good prediction for determining the degree of saturation in both the aeration and saturation zones based on DMT tests. On this basis, the following relationship is proposed:

(19) $$S_{r}=1.50\cdot {\left[\frac{\left(P_{0}-u_{0}\right)\cdot \left(P_{1}-u_{0}\right)}{\sigma_{v0}^{\prime }}\right]}^{-0.33},$$

(20) $$S_{r}=1.50\cdot {\left[\frac{B_{D}}{\sigma_{v0}^{\prime }}\right]}^{-0.33},$$

where the dilatometer pressure number $B_{D}={\left[\left(P_{0}-u_{0}\right)\cdot \left(P_{1}-u_{0}\right)\right]}^{-0.33}$.

## 5. Discussion of the Results

The obtained values of the relative density and degree of saturation for soils from the proposed two new nomogram charts are comparable to those obtained directly after probing from another device or from the calculation methods. The proposed nomograms are convenient and user-friendly, very helpful for both students and designers.

**Figure 15:**
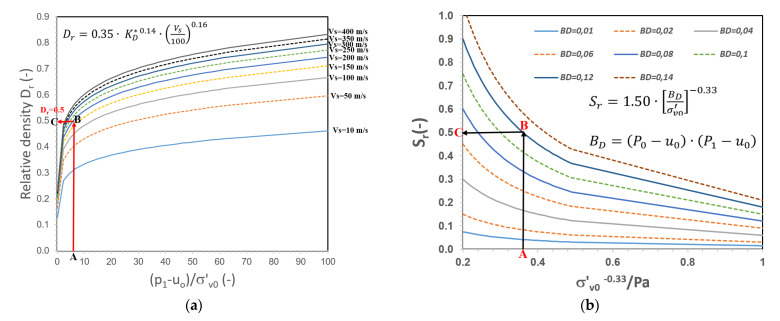
Proposed nomogram charts for determining: (**a**) relative density (*D_r_*) and (**b**) degree of saturation (*S_r_*) based on Marchetti’s dilatometer (DMT).

The nomogram can be used in several ways depending on the data available to the user. With dilatometer pressures *p*_0_ and *p*_1_, *u*_0_, and *σ*_*v*0_ from SDMT values of the tested soil, the approximate relative density and degree of saturation can be read (*procedure: on the nomogram moving from point A to B, then to C, the value at point C is the value of the parameter we are looking for*). The nomograms were used to determine the state of sands in the studied sites, and soils with a granular material (such as sand). The proposed nomogram chart was used to compare the values of the relative density and degree of saturation obtained from the tests by several methods (Figure 15).

The soil type is important for selecting the method of calculating both the degree of compaction (*D_r_*) and the degree of saturation (*S_r_*). Therefore, it is important to become acquainted with the conditions in the course of preparations, e.g., geology, stratigraphy, and hydrology of the study area.

This study was able to propose a formula and a new nomogram for determining parameter *D_r_*, which are regional and allow for obtaining results similar to those from DP tests without distinguishing whether the ground is above or below the groundwater table.

The deviation values obtained in the course of the calculations were acceptable; their values were as follows: a mean square relative deviation (*MSRD*) = 8.0%, and a maximal relative deviation (*MRD*) = 22%.

The obtained formula is as follows: $D_{r}=\alpha_{0}\cdot {\left(\frac{p_{1}-u_{0}}{\sigma_{v0}^{\prime }}\right)}^{\alpha_{1}}\cdot {\left(\frac{V_{s}}{100}\right)}^{\alpha_{2}}$, where *α*_0_ = 0.35, *α*_1_ = 0.14, and *α*_2_ = 0.16.

The performed calculations show that the deviation values were satisfactory for the degree of humidity. The lowest value of 0.02% was obtained for the measurement both below the groundwater table and above the groundwater table; these values were, respectively, a mean square relative deviation (*MSRD*) = 12.0%, and a maximal relative deviation (*MRD*) = 22%. The formula for calculating the degree of saturation that was finally obtained is as follows: $S_{r}=1.50\cdot {\left[\frac{B_{p}}{\sigma_{v0}^{\prime }}\right]}^{-0.33}$, where the dilatometer pressure number $B_{p}=\left(P_{0}-u_{0}\right)\cdot \left(P_{1}-u_{0}\right)$.

## 6. Conclusions

This study presented the results of dynamic probing (DP), time-domain reflectometry (TDR/MUX/MPTS), resistivity cone penetration tests (RCPT), Marchetti dilatometer tests (DMT), and seismic dilatometer tests (SDMT), from which it is possible to develop a relationship to calculate the relative density (*D_r_*) and degree of saturation (*S_r_*) of selected sandy soils. Probing was conducted at five sites (Antoniny, Koszyce, Nielisz, Stegny, and SGGW campus) in Poland.

Based on the results obtained, two relationships were proposed for determining the relative density (*D_r_*) and the degree of saturation (*S_r_*) in mineral soils based on SDMT (DMT). In addition, this paper proposed a new nomogram chart for determining the relative density (*D_r_*) and saturation degree (*S_r_*) from DMT and SDMT tests. The proposed formula and the new nomogram for determining parameter *D_r_* are local in nature. In the future, our study will continue to be devoted to checking the proposed formulas and nomogram charts on other sites at home and abroad.

The proposed nomogram charts may be limited to a greater extent for additional laboratory tests that must be performed to obtain these values. Thanks to the empirical method established, we may reduce the time needed to assess the ground state for non-cohesive soils.

## Figures and Tables

**Table 1:** Index properties of organic soils at the Antoniny, Koszyce, Nielisz, Stegny, and SGGW campus test sites.

Site	Type of Soil	Organic Content *I_om_* (%)	CaCO_3_ Content (%)	Water Content *w_n_* (%)	Liquid Limit *w_L_* (%)	Density	
						Unit Weight of Soil *ρ* (t/m^3^)	Specific Weight of Soil *ρ*_s_ (t/m^3^)
Antoniny	Amorphous Peat	65–75	10–15	310–340	305–450	1.05–1.10	1.45–1.50
	Calcareous Gyttja	5–20	65–90	105–140	80–110	1.25–1.40	2.2–2.30
Koszyce	Amorphous Peat	70–85	5–15	400–550	450	1.05–1.1	1.45–1.50
	Calcareous Gyttja (G_y_)	10–20	65–80	120–160	80–110	1.20–1.35	2.1–2.25
	Calcareous Gyttja (G_y_)	15–20	65–75	180–220	100–110	1.25–1.30	2.2
Nielisz	Organic Mud (M_or_)	20–30	-	120–150	130–150	1.25–1.30	2.25–2.3
	Organic Mud (M_or_)	10–20	-	105–120	110–130	1.30–1.45	2.30–2.40
Stegny	Pliocene Clays	-	-	19.20–28.50	67.6–88.0	2.1–2.2	2.68–2.73
SGGW Campus	Boulder Clay	-	-	5.20–20.10	21.9–26.6	2.0–2.2	2.68–2.73

**Table 2:** Index properties of sandy soils in the Antoniny, Koszyce, and Nielisz embankment test sites, and Stegny and WULS-SGGW campus mineral layer test sites.

Sites	Type of Soil	CaCO_3_	Water Content	Density	
		Content	*w_n_*	Unit Weight of Soil	Specific Weight of Soil
		(%)	(%)	*ρ* (t/m^3^)	*ρ*_s_ (t/m^3^)
Antoniny	sand	<1	6.5	1.7	2.65–2.67
Koszyce		<1	6.2	1.85	2.65–2.67
Nielisz		1–3	6.1	1.85	2.65–2.67
Stegny		<1	5.4	1.7	2.68–2.66
WULS-SGGW Campus		1–3	6.7	1.85	2.68–2.66

## Data Availability

The data cannot be shared because it also belongs to an ongoing research.
